# Supplementary material for: Dopamine control of social novelty preference is constrained by an interpeduncular-tegmentum circuit
Source: Nat Commun. 2024 Apr 3;15:2891. doi: 10.1038/s41467-024-47255-y (PMC10991551; doi:10.1038/s41467-024-47255-y)
Supplement: Supplementary file 1 — Supplementary Information [file 41467_2024_47255_MOESM1_ESM.pdf]

Dopamine control of social novelty preference is constrained by an interpeduncular-tegmentum circuit.

Supplementary Information:

Supplementary Figures 1-9

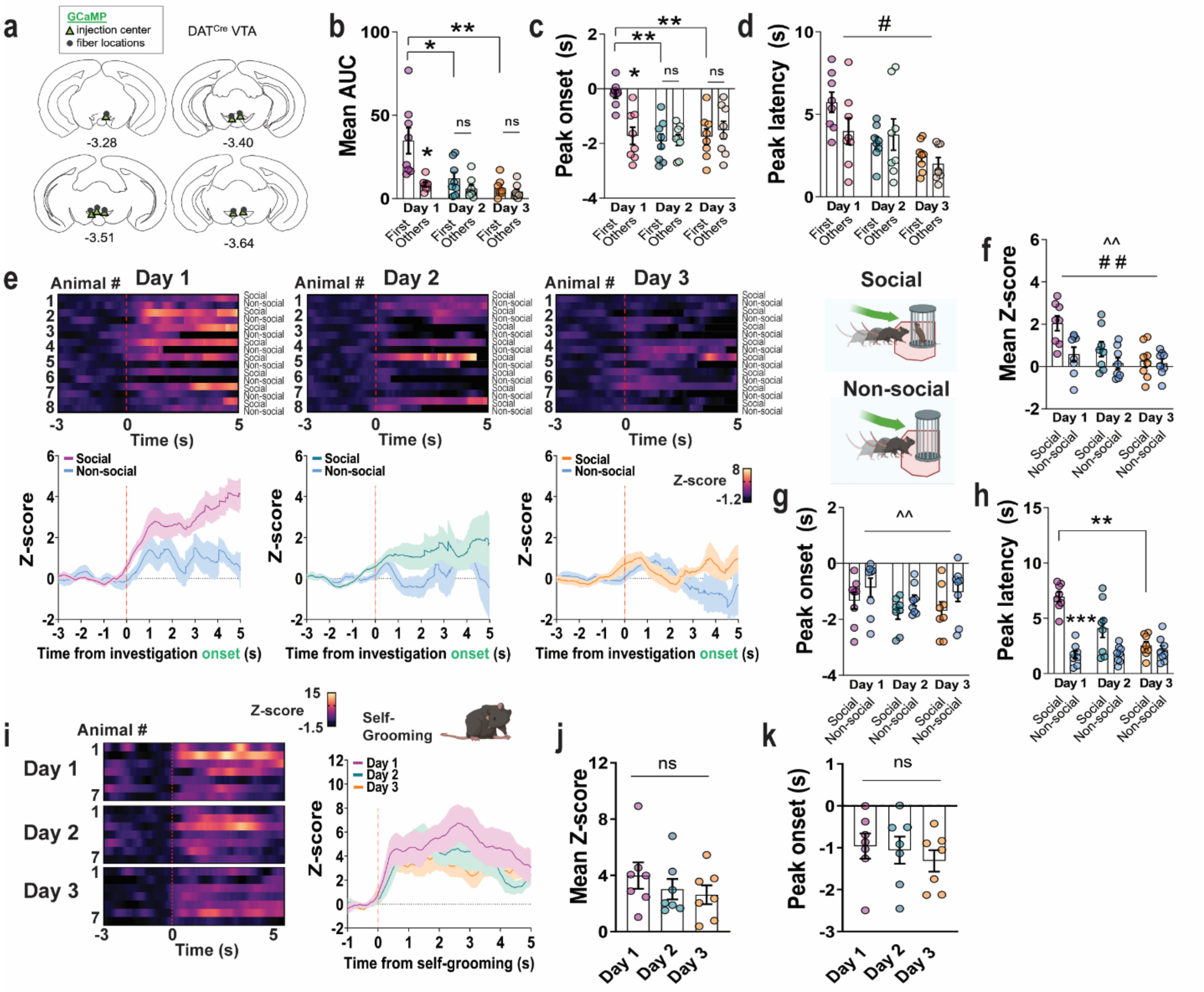

Supplementary Figure 1. VTA DAergic neuronal activity encodes social novelty. (**a**) Fiber placements from VTA DAT^{Cre} recorded animals used in Fig. 1b-h and Fig. 3e-f (n=8 male mice). (**b**) Quantification of activity responses in (Fig. 1d) as mean AUC. Two-way RM ANOVA (time main effect: $F_{(2,14)}$=10.74, P=0.0058; bout main effect: $F_{(1,7)}$=7.299, P=0.0306; interaction: $F_{(2,14)}$=12.19, P=0.0031). (**c**) Peak onset (s) of activity responses in (Fig. 1d). Two-way RM ANOVA (time main effect: $F_{(2,14)}$=6.451, P=0.0172; bout main effect: $F_{(1,7)}$=3.121, P=0.1206; interaction: $F_{(2,14)}$=5.280, P=0.0214). (**d**) Peak latency (s) of activity responses in (Fig. 1d). Two-way RM ANOVA (time main effect: $F_{(2,14)}$=7.110, P=0.0158; bout main effect: $F_{(1,7)}$=1.442, P=0.2688; interaction: $F_{(2,14)}$=1.493, P=0.2617). (**e**) Heatmap representations and *z*-score values of time-locked VTA DAergic activity recordings relative to the onset of investigations (red line) to the social and non-social cylinders on Days 1 to 3. Mouse illustration made with Biorender. (**f**) Quantification of activity responses in (e). Two-way RM ANOVA (social vs non-social main

effect: $F_{(1,14)}$=11.79, P=0.0040; time main effect: $F_{(2,28)}$=6.276, P=0.0085; interaction: $F_{(2,28)}$=2.250, P=0.1241). (**g**) Peak onset (s) of activity responses in (e). Two-way RM ANOVA (social vs non-social main effect: $F_{(1,14)}$= 9.959, P=0.007; time main effect: $F_{(2,28)}$=1.003, P=0.3699; interaction: $F_{(2,28)}$=0.0710, P=0.9316). (**h**) Peak latency (s) of activity responses in (e). Two-way RM ANOVA (social vs non-social main effect: $F_{(1,14)}$= 39.88, P<0.0001; time main effect: $F_{(2,28)}$=10.04, P=0.0013; interaction: $F_{(2,28)}$=14.28, P<0.0001). (**i**) Heatmap representations and *z*-score values of time-locked VTA DAergic activity recordings relative to the onset of self-grooming events (red line) during the social test on Days 1 to 3. Mouse illustration made with Biorender. (**j**) Quantification of activity responses in (i). One-way RM ANOVA ($F_{(2,12)}$=0.8147, P=0.4398). (**k**) Peak onset (s) of activity responses in (i). One-way RM ANOVA ($F_{(2,12)}$=0.4445, P=0.5818). Data represent mean ± SEM. Two-way RM ANOVA ^^p<0.01, #p<0.05, ##p<0.01. Šidák's multiple comparisons *p<0.05, ** p<0.01, *** p<0.001. represent mean ± SEM. Two-way RM ANOVA ^^p<0.01, #p<0.05, ##p<0.01. Šidák's multiple comparisons *p<0.05, ** p<0.01, *** p<0.001. Source data are provided as a Source Data file.

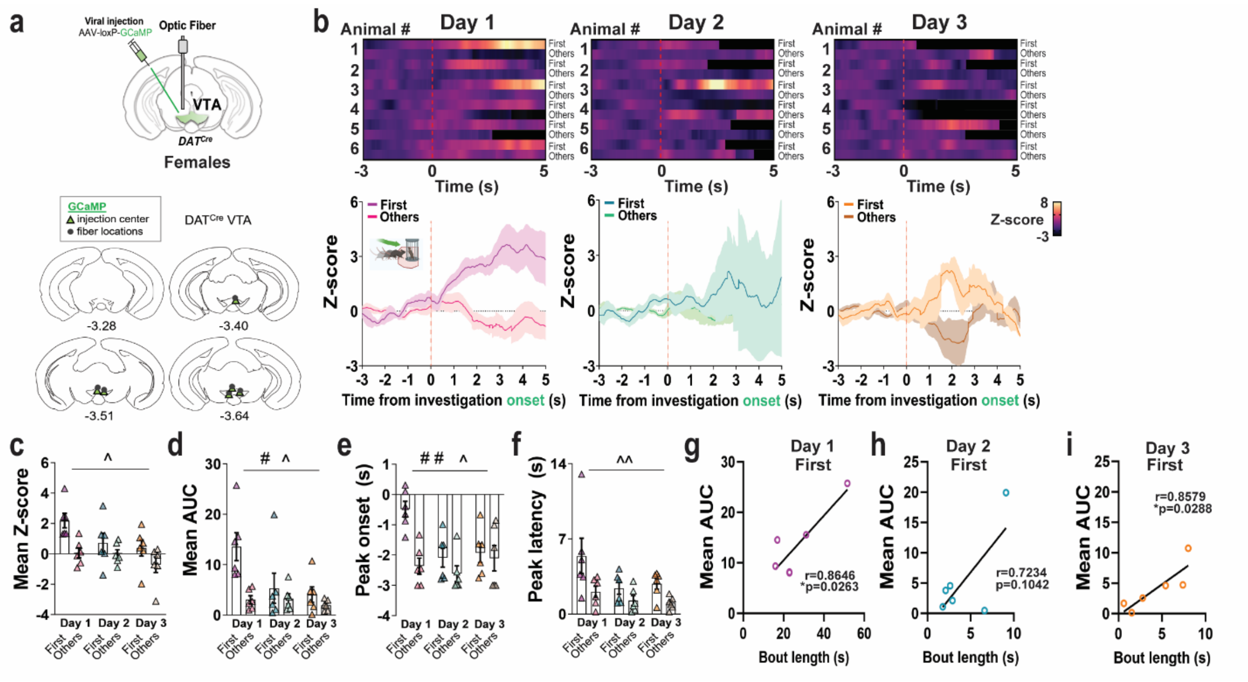

Supplementary Figure 2. Social novelty increases VTA DAergic neuronal activity in female mice. (**a**) Schematic of the viral injection and recording strategy used in DAT^{Cre} female mice (*top*) and fiber placements from VTA DAT^{Cre} recorded females (n = 6 mice) (*bottom*). (**b**) Heatmap representations and *z*-score values of time-locked VTA DAergic activity recordings relative to the time initiating a social investigation (red line) on Days 1 to 3 of the social test. The first social investigation is compared to the subsequent ones. Mouse illustration made with Biorender. Quantification of activity responses in (b) as (**c**) mean z-score, Two-way RM ANOVA (time main effect: $F_{(2,10)}$=4.656, *P*=0.0652; bout main effect: $F_{(1,5)}$=9.548, *P*=0.0272; interaction: $F_{(2,10)}$=1.528, *P*=0.2673) and (**d**) mean AUC, Two-way RM ANOVA $F_{(2,10)}$=5.285, *P*=0.0280; bout main effect: $F_{(1,5)}$=9.878, *P*=0.0256; interaction: $F_{(2,10)}$=3.381, *P*=0.0772). (**e**) Peak onset (s) of activity, two-way RM ANOVA (time main effect: $F_{(2,10)}$= 11.23, *P*=0.0056; bout main effect: $F_{(1,5)}$=7.371, *P*=0.0420; interaction: $F_{(2,10)}$=2.833, *P*=0.1191). (**f**) Peak latency (s) of activity responses, two-way RM ANOVA (time main effect: $F_{(2,10)}$= 2.727, *P*=0.1551; bout main effect: $F_{(1,5)}$=46.81, *P*=0.0010; interaction: $F_{(2,10)}$=0.8800, *P*=0.4213). Correlation between the bout length duration (s) of social investigations and the mean area under the curve (AUC) of the first VTA GCaMP signal on (**g**) Day 1, Two-tailed Pearson r (r=0.8646, R^2=0.7474, p=0.0263), (**h**) Day 2, Two-tailed Pearson r (r=0.7234, R^2=0.5233, p=0.1042), and (**i**) Day 3, Two-tailed Pearson r (r=0.8579, R^2=0.7360, p=0.0288) of the social paradigm. Data represent mean ± SEM. Two-way

RM ANOVA ^ $p<0.05$, ^^ $p<0.01$, # $p<0.05$, ## $p<0.01$. Source data are provided as a Source Data file.

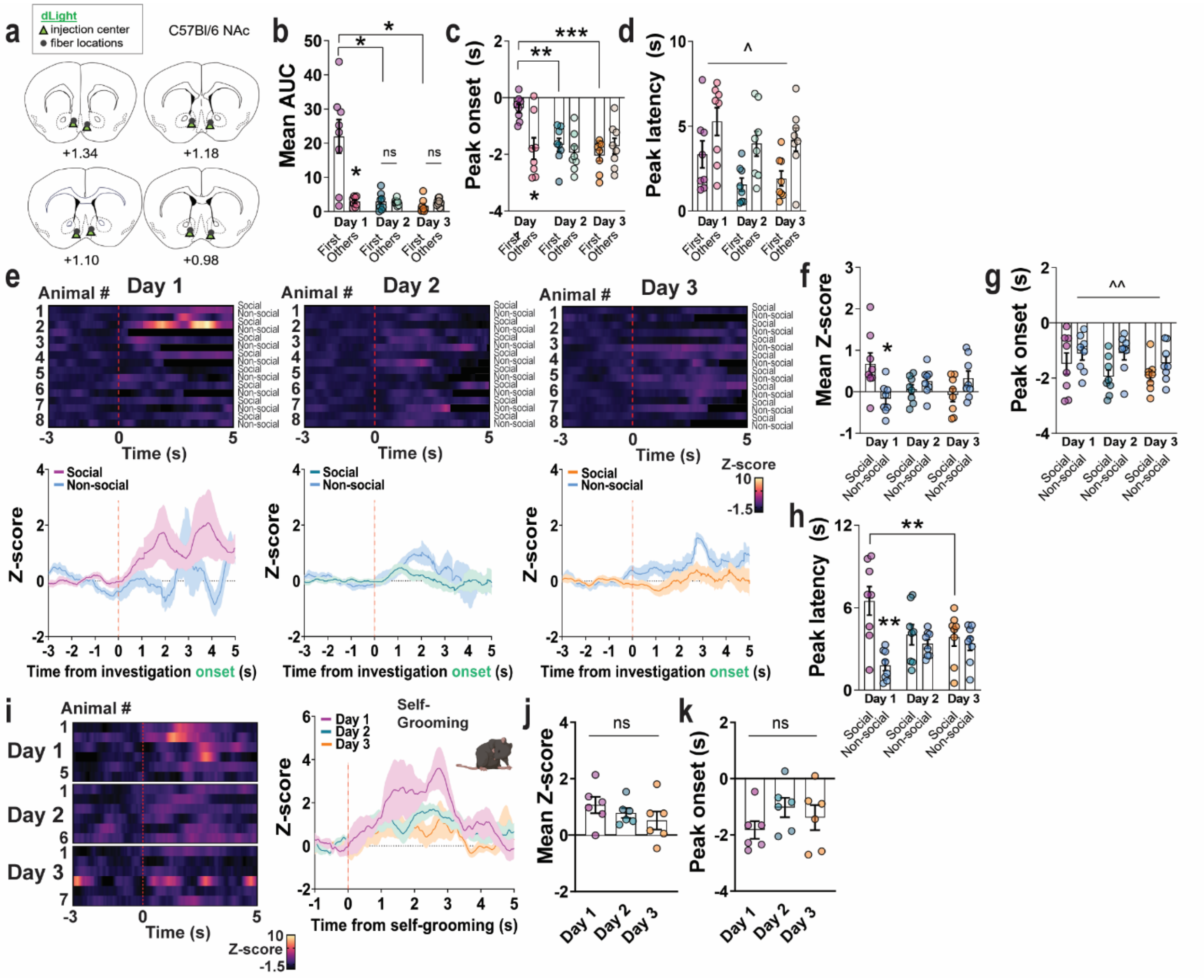

Supplementary Figure 3. NAc DA activity responds to social novelty and self-grooming.

(**a**) Schematic of fiber placements from the recorded dLight 1.2. NAc C57BL/6 animals used in Fig. 2c-g (n=8 male mice). (**b**) Quantification of activity responses in (Fig. 2c) as mean AUC. Two-way RM ANOVA $F_{(2,14)}$=13.87, P=0.0060; bout main effect: $F_{(1,7)}$=12.96, P=0.0087; interaction: $F_{(2,14)}$=14.34, P=0.0052). (**c**) Peak onset (s) of activity responses in (Fig. 2c). Two-way RM ANOVA (time main effect: $F_{(2,14)}$= 6.804, P=0.0313; bout main effect: $F_{(1,7)}$=3.577, P=0.1005; interaction: $F_{(2,14)}$=8.671, P=0.0041). (**d**) Peak latency (s) of activity responses in (Fig. 2c). Two-way RM ANOVA (time main effect: $F_{(2,14)}$= 3.086, P=0.0970; bout main effect: $F_{(1,7)}$=9.404, P=0.0182; interaction: $F_{(2,14)}$=0.1056, P=0.8980). (**e**) Heatmap representations and *z*-score values of time-locked NAc DA signals relative to the onset of investigations (red line) to the social and non-social cylinders on Days 1 to 3 of the social test. (**f**) Quantification of activity responses in (e). Two-way RM ANOVA (social vs non-social main effect: $F_{(1,14)}$=0.9053,

P=0.3575; time main effect: $F_{(2,28)}$=0.2588, P=0.7562; interaction: $F_{(2,28)}$=6.162, P=0.0061). (**g**) Peak onset (s) of activity responses in (e). Two-way RM ANOVA (social vs non-social main effect: $F_{(1,14)}$=11.88, P=0.0039; time main effect: $F_{(2,28)}$=0.8863, P=0.4226; interaction: $F_{(2,28)}$=0.3963, P=0.6765). (**h**) Peak latency (s) of activity responses in (e). Two-way RM ANOVA (social vs non-social main effect: $F_{(1,14)}$=6.469, P=0.0234; time main effect: $F_{(2,28)}$=0.8072, P=0.4342; interaction: $F_{(2,28)}$=14.16, P<0.0001).(**i**) Heatmap representations and z-score values of time-locked NAc DA signals relative to the onset of self-grooming events (red line) during the social test on Days 1 to 3. Mouse illustration made with Biorender. (**j**) Quantification of activity responses in (i). One-way RM ANOVA ($F_{(2,10)}$=0.7499, P=0.4746). (**k**) Peak onset (s) of activity responses in (i). One-way RM ANOVA ($F_{(2,10)}$=1.172, P=0.3484). Data represent mean ± SEM. Two-way RM ANOVA ^p<0.05, ^^p<0.01. Šidák's multiple comparisons *p<0.05, ** p<0.01, *** p<0.001. Source data are provided as a Source Data file.

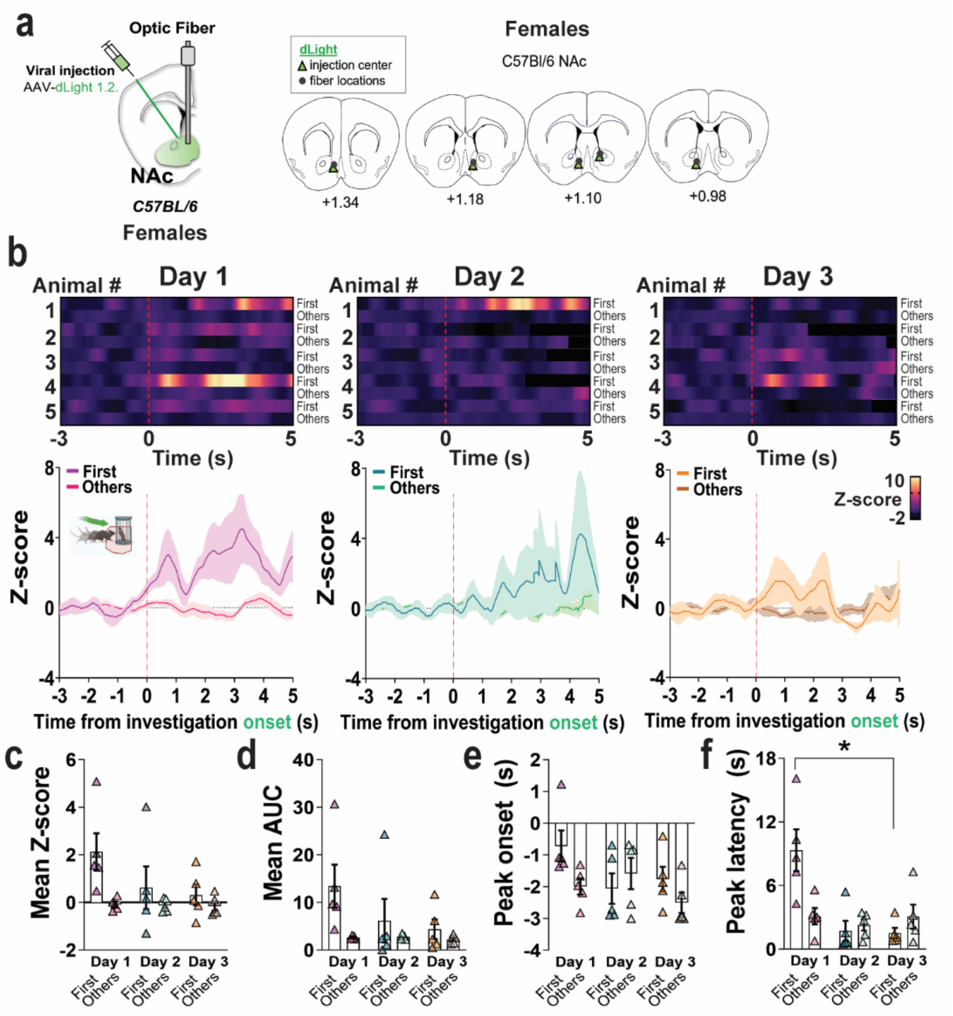

Supplementary Figure 4. Social novelty increases NAc DA release in female mice.

(**a**) Schematics of viral injection and recording strategy used in C57BL/6 female mice to measure NAc DA signals (*left*) and fiber placements from the recorded dLight 1.2. NAc C57BL/6 females (n=5 mice)(*right*). (**b**) Heatmap representations and *z*-score values of time-locked NAc DA signals relative to the time initiating a social investigation (red line) on Days 1 to 3 of the social test. The first social investigation is compared to the subsequent ones. Mouse illustration made with Biorender. Quantification of activity responses in (b) as (**c**) mean z-score, two-way RM ANOVA (time main effect: $F_{(2,8)}$=1.855, P=0.2235; bout main effect: $F_{(1,4)}$=4.459, P=0.1023; interaction: $F_{(2,8)}$=2.251, P=0.1996), (**d**) mean AUC, two-way RM ANOVA (time main effect: $F_{(2,8)}$=1.731, P=0.2486; bout main effect: $F_{(1,4)}$=4.433, P=0.1030; interaction: $F_{(2,8)}$=1.756, P=0.2486). (**e**) Peak onset (s) of activity responses in (b). Two-way RM ANOVA (time main effect: $F_{(2,8)}$=2.084, P=0.1920; bout main effect: $F_{(1,4)}$=4.900, P=0.0913; interaction: $F_{(2,8)}$=2.001, P=0.2099) . (**f**) Peak

latency (s) of activity responses in (b). Two-way RM ANOVA (time main effect: $F_{(2,8)}$=12.73, P=0.0138; bout main effect: $F_{(1,4)}$=4.058, P=0.1142; interaction: $F_{(2,8)}$=7.050, P=0.0287). Šidák's multiple comparisons *$p<0.05$. Data represent mean ± SEM. Source data are provided as a Source Data file.

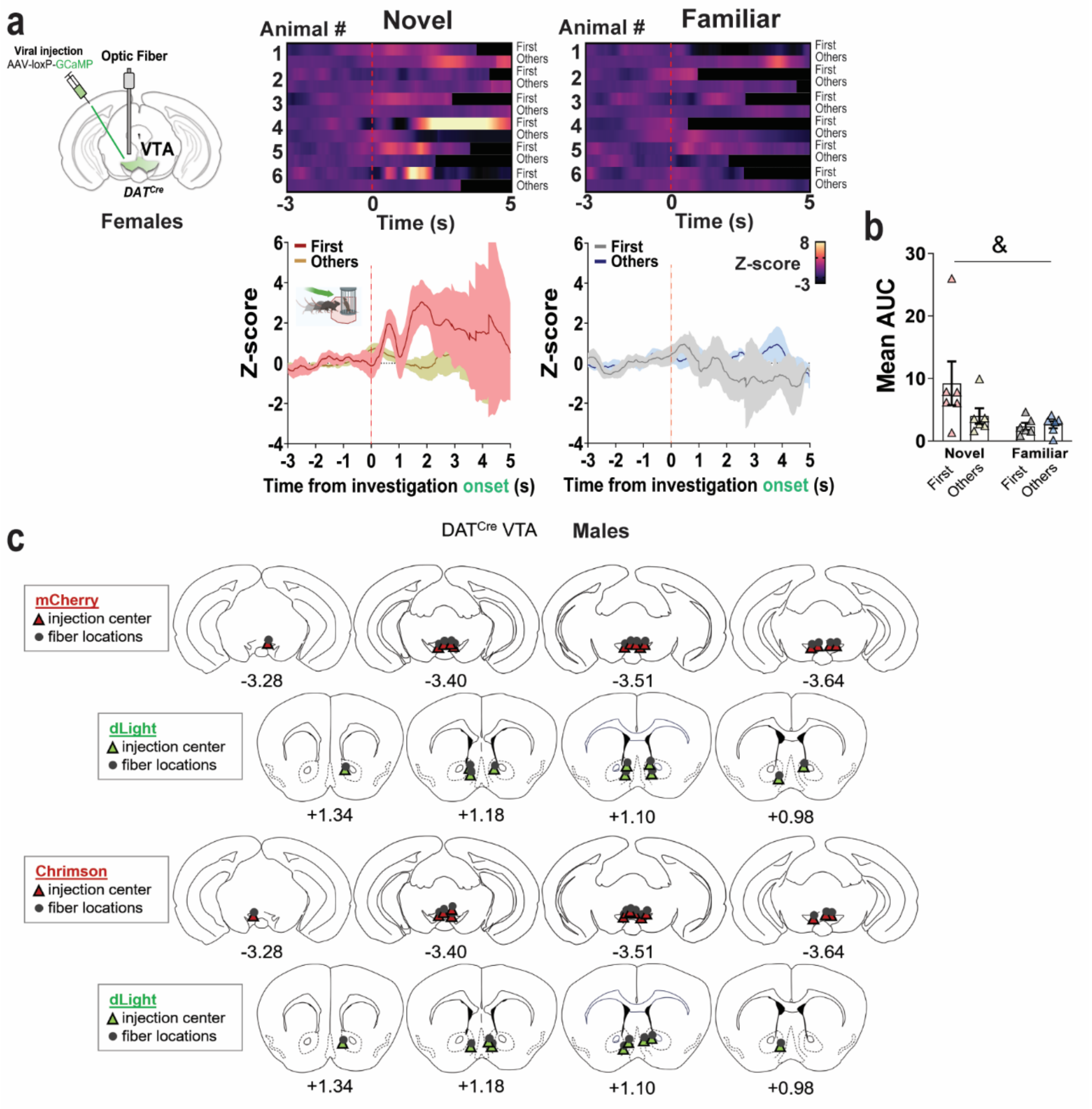

Supplementary Figure 5. VTA DA activity increases in female mice with social novelty investigations during NP. (**a**) *Left*, schematic of viral injection and recording strategy used to measure female VTA DA GCaMP. *Right*, heatmap and *z*-scores of DA VTA GCaMP signals time-locked to novel and familiar investigations in the social NP test (n = 6 female mice). Mouse illustration made with Biorender. (**b**) Quantification of responses in (a). Two-way RM ANOVA (novel vs familiar main effect: $F_{(1,10)}$=5.769, P=0.0372; bout main effect: $F_{(1,10)}$=1.451, P=0.2562; interaction: $F_{(1,10)}$=1.779, P=0.2119). (**c**) Viral injections and fiber placements in $DAT^{VTA:mCherry}$ and $DAT^{VTA:Chrimson}$ animals used in Fig. 3j-n (n = 14 male mice/group). Data represent mean ± SEM. Two-way RM ANOVA & p<0.05. Source data are provided as a Source Data file.

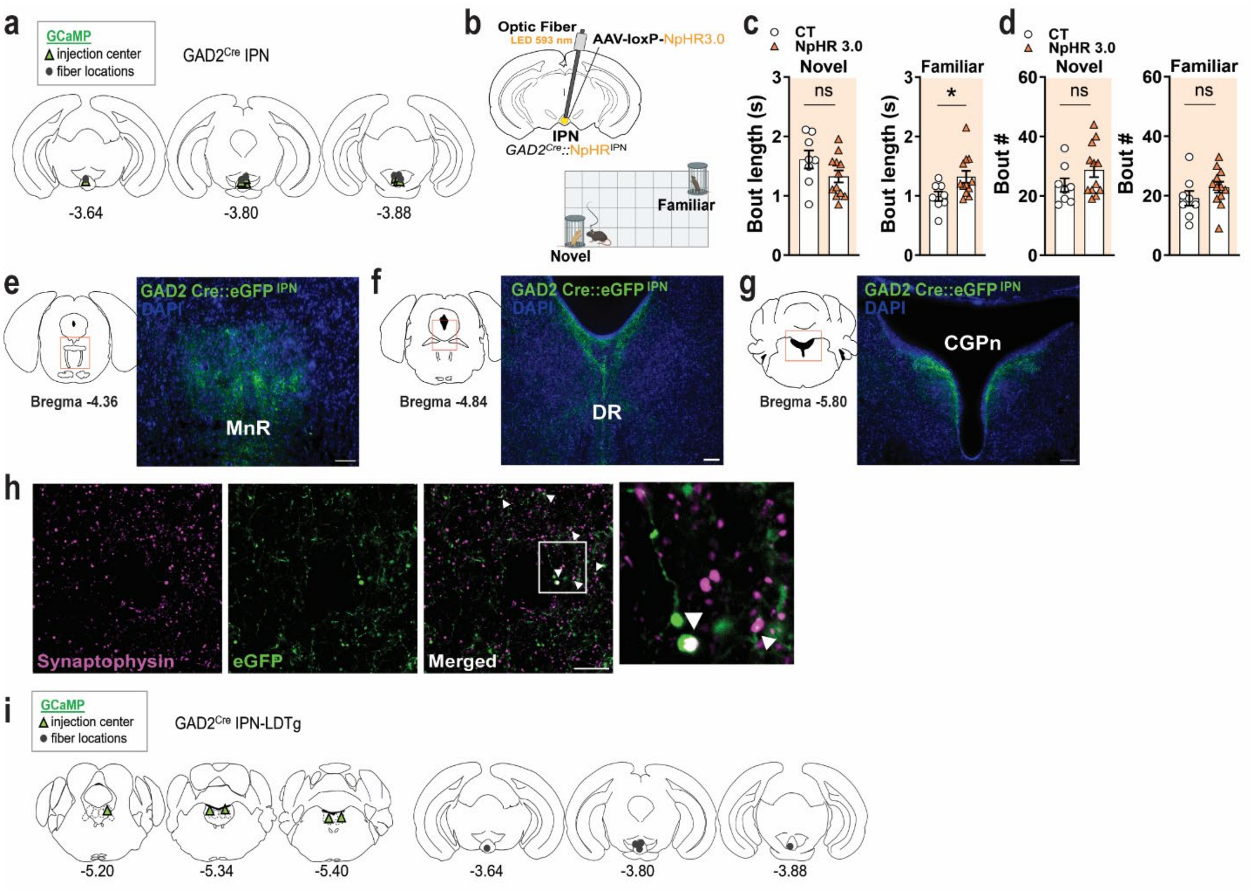

Supplementary Figure 6. IPN GAD2 neurons control bout length of familiar social investigations and send projections to midbrain/hindbrain areas. (**a**) Schematic of fiber placements from recorded $GAD2^{Cre}$ animals expressing GCaMP in the IPN used in Fig. 4a-f (n=8 male mice). (**b**) Schematics of NpHR3.0 viral-mediated expression in the IPN of $GAD2^{Cre}$ mice and the NP behavioral paradigm. (**c**) Bout length (s) of social investigations in control (CT) and $GAD2^{IPN:NpHR}$ mice during the NP test (n=8-12 male mice/group). *Left*, novel investigations unpaired two-tailed t-test ($t_{(18)}$=1.637, P=0.119). *Right*, familiar investigations unpaired two-tailed t-test ($t_{(18)}$=2.348, P=0.0305). (**d**) Bout number of novel and familiar social investigations during the NP test in CT and $GAD2^{IPN:NpHR}$ mice. *Left*, novel investigations unpaired two-tailed t-test ($t_{(18)}$=1.443, P=0.1662). *Right*, familiar investigations unpaired two-tailed t-test ($t_{(18)}$=1.228, P=0.2354). Axonal projections from eGFP-expressing IPN GAD2 neurons innervating the median raphe (MnR) (**e**), the dorsal raphe (DR) (**f**) and the central pontine gray (CGPn)(**g**). Nuclei are counterstained with DAPI. Scale bars 100µm. (**h**) Immunostaining of the presynaptic marker synaptophysin (magenta) in the LDTg, colocalized (arrows) with eGFP+ axon terminals from IPN GAD2 neurons (green). Scale bar 50 µm. (**i**) Schematics of retroviral GCaMP injection location in

the LDTg and fiber placements in the IPN from IPN→LDTg GAD2 recorded animals used in Fig. 5a-f (n = 5 male mice). Data represent mean ± SEM. Unpaired two-tailed t-test *$p<0.05$. Source data are provided as a Source Data file.

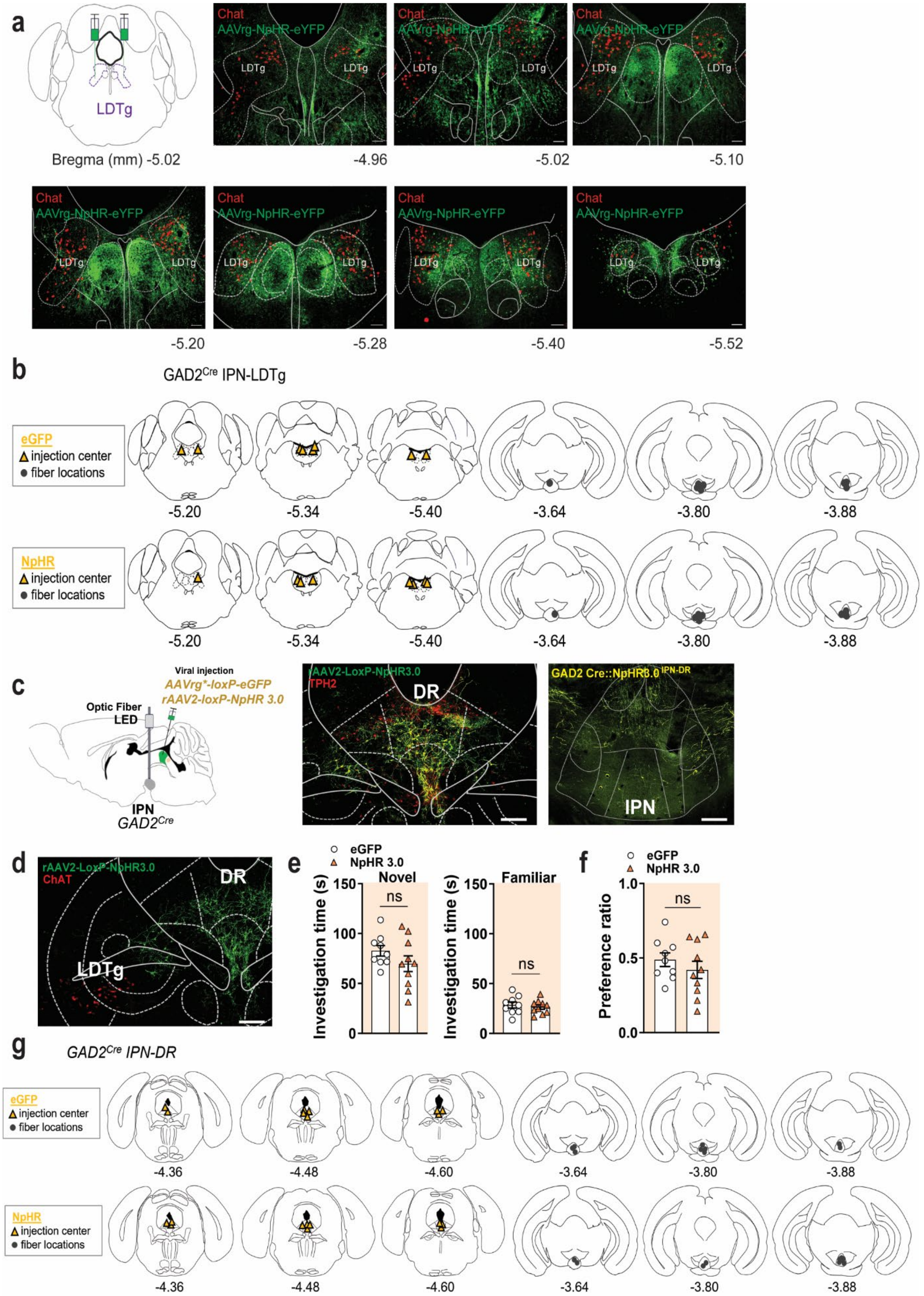
a
LDTg
Bregma (mm) -5.02
Chat
AAVrg-NpHR-eYFP
-4.96
-5.02
-5.10
-5.20
-5.28
-5.40
-5.52
b
GAD2Cre IPN-LDTg
eGFP
injection center
fiber locations
NpHR
-5.20
-5.34
-5.40
-3.64
-3.80
-3.88
c
Viral injection
AAVrg*-loxP-eGFP
rAAV2-loxP-NpHR 3.0
Optic Fiber
LED
IPN
GAD2Cre
rAAV2-LoxP-NpHR3.0
TPH2
DR
GAD2 Cre::NpHR3.0 IPN-DR
d
ChAT
e
eGFP
NpHR 3.0
Novel
Familiar
Investigation time (s)
ns
f
Preference ratio
g
GAD2Cre IPN-DR
-4.36
-4.48
-4.60

Supplementary Figure 7. Social NP ratio is not mediated by IPN GAD2 neurons projecting to the DR. (**a**) Schematics of viral injection used and representative images of retroviral-mediated NpHR injection at different Bregma coordinates containing the LDTg in $GAD2^{Cre}$ mice. ChAT immunostaining (red) identifies cholinergic neurons in the LDTg area. Scale bars, 100µm. (**b**) Schematics of viral injections and fiber placements in $GAD2^{IPN\text{-}LDTg:eGFP}$ and $GAD2^{IPN\text{-}LDTg:NpHR}$ animals used in Fig. 5g-m (n = 8 male mice/group). (**c**) *Left*, diagram of the viral injection strategy used. *Middle*, representative image of retroviral-mediated NpHR injection in the DR of $GAD2^{Cre}$ mice. Tph2 (red) immunostaining defines the DR area. Scale bars, 100µm. *Right*, representative image of viral expression and fiber placement in the IPN of $GAD2^{IPN\text{-}DR:NpHR}$ animals. Scale bars, 100µm. (**d**) Representative image of retroviral-mediated NpHR injection in the DR of $GAD2^{Cre}$ mice with ChAT immunostaining (red). Scale bars, 100µm. (**e**) Time (s) of novel and familiar social investigations in $GAD2^{IPN\rightarrow DR:eGFP}$ and $GAD2^{IPN\rightarrow DR:NpHR}$ mice during the NP task. (n= 9-10 male mice/group). *Left*, novel investigations unpaired two-tailed t-test ($t_{(17)}$=1.329, P=0.2013). *Right*, familiar investigations unpaired two-tailed t-test ($t_{(17)}$=0.5588, P=0.5836). (**f**) Social NP ratio in $GAD2^{IPN\rightarrow DR:eGFP}$ and $GAD2^{IPN\rightarrow DR:NpHR}$ mice. Unpaired two-tailed t-test ($t_{(17)}$=0.9105, P=0.3753). (**g**) Schematics of viral injection and optic fiber placement in the animals used in (e-f). Data represent mean ± SEM. Source data are provided as a Source Data file.

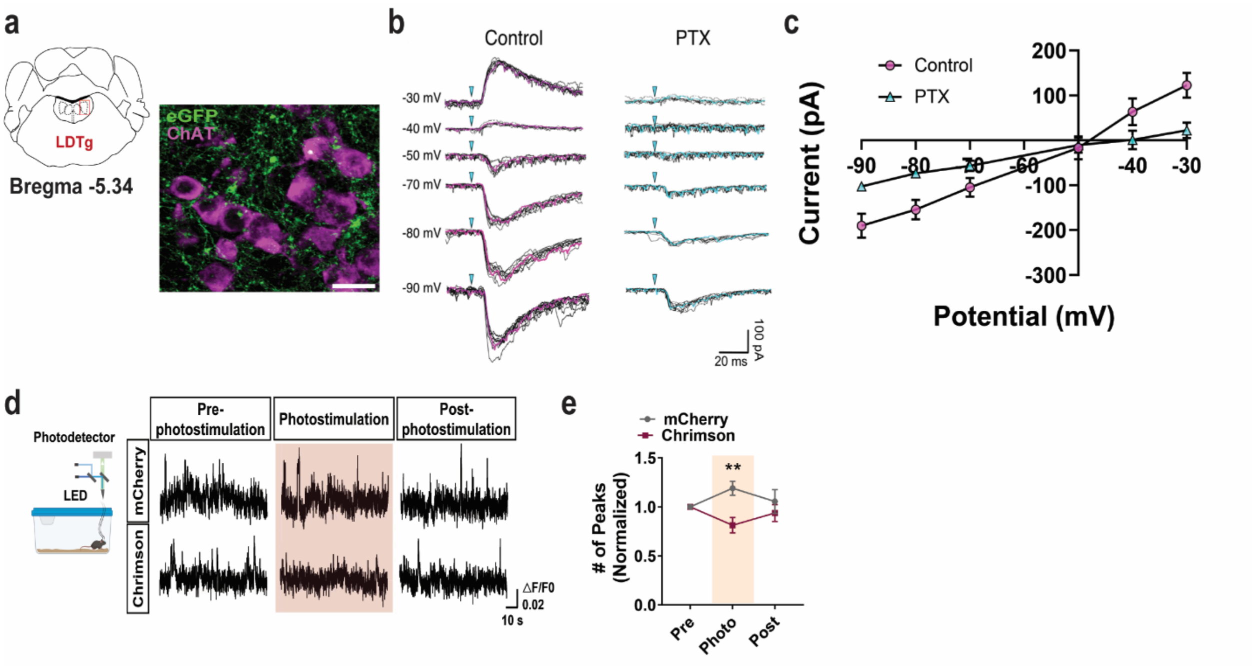

Supplementary Figure 8. The IPN→LDTg circuit innervates Chat+ LDTg→VTA neurons and regulate the release of DA in the NAc. (**a**) eGFP-expressing IPN GAD2+ axons (green) surrounding cholinergic neurons (ChAT immunostaining, magenta) in the LDTg area. Scale bar 50 µm. (**b**) Representative traces of LDTg→VTA ChAT+ neuronal responses upon blue light photostimulation (450 nm, blue arrow) of IPN axon terminals in control (*left*) and during exposure to picrotoxin (PTX, 50µM)(*right*) conditions at holding potentials of -30, -40, -50, -70, -80, and -90 mV. At each potential, we recorded 10 traces (black) with averages represented in magenta (control) and turquoise (PTX) lines. In both groups, LDtg neurons have a reversal potential of approximately -45 mV. (**c**) Current-voltage relationship of light-evoked currents in control conditions and in the presence of PTX (n = 3 animals, 6 cells). (**d**) Representative traces of NAc DA signals (dF/F0) in $GAD2^{IPN \rightarrow LDTg:mCherry}$ and $GAD2^{IPN \rightarrow LDTg:Chrimson}$ mice in their home cages pre-photostimulation, during photostimulation and post-photostimulation of the IPN^{GAD2}→LDTg circuit. (**e**) Normalized number of NAc DA peak activity in $GAD2^{IPN \rightarrow LDTg:mCherry}$ and $GAD2^{IPN \rightarrow LDTg:Chrimson}$ mice in their home cages during a 5-min recording period pre-photostimulation, during photostimulation and post-photostimulation of the IPN^{GAD2}→LDTg circuit. Two-way RM ANOVA (virus main effect: $F_{(1,8)}$=6.360, P=0.0357; time main effect: $F_{(2,16)}$=0.003236, P=0.9968; interaction: $F_{(2,16)}$=3.649, P=0.0495) (n = 5 male mice/group). Šidák's multiple comparisons ** p<0.01. Data represent mean ± SEM. Source data are provided as a Source Data file.

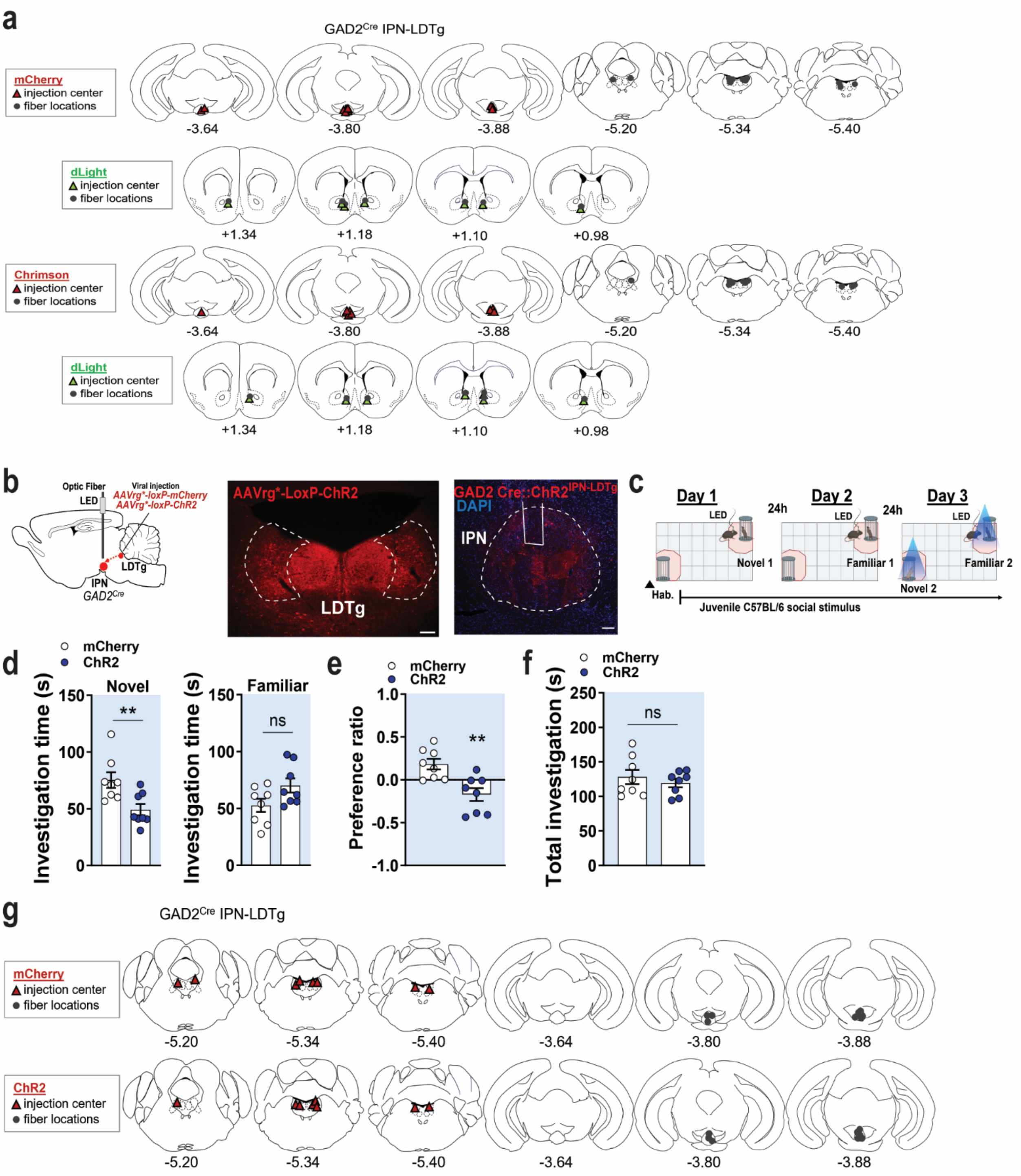

Supplementary Figure 9. Optogenetic manipulation of the IPN→LDTg GAD2 neuronal circuit during the social NP test. (**a**) Schematics of viral injection and fiber placement in GAD2$^{IPN→LDTg:mCherry}$ and GAD2$^{IPN→LDTg:Chrimson}$ mice during the NP task (n = 10-11 male mice/group) used in Fig. 7. (**b**) *Left,* schematics of the retroviral injection and optic fiber implant strategy used. *Middle*, representative image of retroviral-mediated ChR2:mCherry injection in the

LDTg of GAD2^{Cre} mice. *Right*, photomicrograph of retroviral ChR2:mCherry-expressing IPN→LDTg neurons and fiber track in the IPN. Nuclei are counterstained with DAPI. Scale bars 100μm. (**c**) Schematic of the social paradigm, generated using Biorender, used with closed-loop optogenetic stimulation during the NP task. (**d**) Time (s) of novel and familiar social investigations in GAD2$^{IPN \rightarrow LDTg:mCherry}$ and GAD2$^{IPN \rightarrow LDTg:ChR2}$ mice during the NP task. (n= 8 male mice/group). *Left*, novel investigations unpaired two-tailed t-test ($t_{(14)}$=3.084, P=0.0081). *Right*, familiar investigations unpaired two-tailed t-test ($t_{(14)}$=2.033, P=0.0614). (**e**) Social NP ratio in GAD2$^{IPN \rightarrow LDTg:mCherry}$ and GAD2$^{IPN \rightarrow LDTg:ChR2}$ mice. Unpaired two-tailed t-test ($t_{(14)}$=3.652, P=0.026). (**f**) Total time (s) in GAD2$^{IPN \rightarrow LDTg:mCherry}$ and GAD2$^{IPN \rightarrow LDTg:ChR2}$ mice during the NP task. Unpaired two-tailed t-test ($t_{(14)}$=0.7392, P=0.4720). (**g**) Schematics of viral injection and fiber placement in GAD2$^{IPN \rightarrow LDTg:mCherry}$ and GAD2$^{IPN \rightarrow LDTg:ChR2}$ mice used in (c-f). Data represent mean ± SEM. Unpaired two-tailed t-test **p<0.01. Source data are provided as a Source Data file.
